# The Purine-Utilizing Bacterium *Clostridium acidurici* 9a: A Genome-Guided Metabolic Reconsideration

**DOI:** 10.1371/journal.pone.0051662

**Published:** 2012-12-11

**Authors:** Katrin Hartwich, Anja Poehlein, Rolf Daniel

**Affiliations:** Department of Genomic and Applied Microbiology, and Göttingen Genomics Laboratory, Institute of Microbiology and Genetics, Georg-August University Göttingen, Göttingen, Germany; Belgian Nuclear Research Centre SCK/CEN, Belgium

## Abstract

*Clostridium acidurici* is an anaerobic, homoacetogenic bacterium, which is able to use purines such as uric acid as sole carbon, nitrogen, and energy source. Together with the two other known purinolytic clostridia *C. cylindrosporum* and *C. purinilyticum*, *C. acidurici* serves as a model organism for investigation of purine fermentation. Here, we present the first complete sequence and analysis of a genome derived from a purinolytic *Clostridium*. The genome of *C. acidurici* 9a consists of one chromosome (3,105,335 bp) and one small circular plasmid (2,913 bp). The lack of candidate genes encoding glycine reductase indicates that *C. acidurici* 9a uses the energetically less favorable glycine-serine-pyruvate pathway for glycine degradation. In accordance with the specialized lifestyle and the corresponding narrow substrate spectrum of *C. acidurici* 9a, the number of genes involved in carbohydrate transport and metabolism is significantly lower than in other clostridia such as *C. acetobutylicum*, *C. saccharolyticum*, and *C. beijerinckii*. The only amino acid that can be degraded by *C. acidurici* is glycine but growth on glycine only occurs in the presence of a fermentable purine. Nevertheless, the addition of glycine resulted in increased transcription levels of genes encoding enzymes involved in the glycine-serine-pyruvate pathway such as serine hydroxymethyltransferase and acetate kinase, whereas the transcription levels of formate dehydrogenase-encoding genes decreased. Sugars could not be utilized by *C. acidurici* but the full genetic repertoire for glycolysis was detected. In addition, genes encoding enzymes that mediate resistance against several antimicrobials and metals were identified. High resistance of *C. acidurici* towards bacitracin, acriflavine and azaleucine was experimentally confirmed.

## Introduction

Clostridia represent one of the largest and most heterogeneous classes within the bacteria [1]. The genus *Clostridium* belongs to the phylum Firmicutes and its members share a Gram-positive cell wall and an anaerobic lifestyle. Special metabolic traits have been found in clostridia such as the Stickland reaction in *C. sporogenes* [2] or *C. sticklandii* [3] and different routes for fermentation of purines, which are dependent on the availability of certain trace elements [4], [5]. *C. acidurici*, *C. cylindrosporum* and *C. purinilyticum* are the described representatives of purinolytic clostridia, which are able to use purines as sole carbon, nitrogen, and energy source [5]–[7]. *C. acidurici* was discovered in 1909 by Liebert and originally named “*Bacillus acidi-urici*” [7]. The strain was isolated from garden soil in a medium containing uric acid and K_2_HPO_4_ dissolved in tap water. Liebert observed an active motile, spore-forming, and rod-shaped bacterium that was able to convert uric acid to ammonia, carbon dioxide, and acetate. In the late 1930`s, Barker started to work on uric acid-fermenting anaerobes and rediscovered and reclassified this organism as *Clostridium acidi-urici*. The type strain 9a was defined in 1942 [6]. Interestingly, it could be isolated from nearly all soil samples but also from avian feces indicating that this organism plays a role in decomposition of uric acid, which is the main nitrogenous end product of avians [7]. Most purines can be fermented by *C. acidurici* but sugars, amino acids or complex nitrogenous compounds such as tryptone are not degraded. Nevertheless, it is able to synthesize all amino acids *de novo.* For purinolytic clostridia, two pathways forming acetate from purines are known. (i) The glycine-serine-pyruvate pathway in which glycine is converted to serine and then to acetate via pyruvate and acetyl phosphate by the activities of the glycine cleavage complex and formate dehydrogenase [8]. (ii) The energetically favorable glycine reductase pathway [4] in which formate dehydrogenase and the glycine cleavage complex are still involved. In this pathway, acetate is synthesized directly by the reduction of glycine via the selenium-dependent enzyme glycine reductase. The use of the glycine reductase pathway has been postulated for all three purinolytic clostridia, although reductase activity has not been detected in *C. acidurici* [4]. In this study, a genome-based approach was employed to solve such inconsistencies. Although the fascinating metabolism of purinolytic clostridia was subject of many investigations [4], [9]–[11], genome-wide studies of these organisms have not been published. Here we present the first completely sequenced and annotated genome of the purinolytic *C. acidurici* type strain 9a. Based on a comparative genome analysis, we performed a genome-guided physiological analysis of *C. acidurici* 9a and provide a general overview of the metabolic capabilities of this organism.

## Materials and Methods

### Strains and Growth Conditions

The type strain *Clostridium acidurici* 9a (DSM 604) was obtained from the DSMZ (German Collection of Microorganisms and Cell Cultures, Braunschweig, Germany). Cultivation was performed in liquid uric acid medium (pH 7.3) containing 12 mM uric acid, 12 mM KOH, 4 mM K_2_HPO_4_, 1 g/l yeast extract, 0.14 mM MgSO_4_ x 7H_2_O, 6.3 µM FeSO_4_ x 7H_2_O, 29 µM CaCl_2_ x 2H_2_O, 0.1 µM MnSO_4_ x H_2_O, 0.1 µM Na_2_SeO_3_ x 5H_2_O, 0.1 µM Na_2_WO_4_ x 2H_2_O, 0.1 µM Na_2_MoO_4_ x 2H_2_O, 4.4 µM resazurin, 20 mM KHCO_3_, and 29.2 mM thioglycolic acid [10]. Cells were grown at 37°C under anaerobic conditions according to the method of Rabinowitz [12]. For growth tests with substrate variations, the medium was altered by lowering the uric acid concentration to 10 mM, 5 mM, 3 mM or 0 mM, and/or the addition of 100 mM glycine. Growth tests were performed in 10 ml medium, which was inoculated to an optical density (OD) of 0.1 using an overnight-grown culture of *C. acidurici* 9a. OD was determined spectrophotometrically at 600 nm (OD_600 nm_) with a WPA CO8000 Biowave cell density meter (Biochrom Ltd, Cambridge, UK). All tests were carried out in triplicate.

### Genome Sequencing and Finishing

Genome sequencing of *C. acidiurici* 9a was done using 454 Titanium pyrosequencing technology as recommended by the manufacturer (Roche, Penzberg, Germany). Raw sequences were assembled into contigs using the Newbler assembly tool v2.3 from Roche. Gap closure and all manual editing was done using the software Gap4 (v 4.11) of the Staden package [13]. The contig order was determined by employing vectorette PCR [14] and multiplex PCR [15] approaches. Remaining gaps were closed by PCR-based approaches and Sanger sequencing [16] of the resulting PCR products using Big Dye 3.0 chemistry and an ABI3730XL capillary sequencer (Applied Biosystems, Life Technologies GmbH, Darmstadt, Germany).

### Gene Prediction and Annotation

Initial gene prediction was done using the YACOP tool [17]. All predicted genes were manually curated based on GC frame plot analysis, presence of ribosome-binding sites, and comparison to known protein-encoding sequences employing the Sanger Artemis tool [18]. Functional annotation was initially done with the ERGO software tool [19] (Integrated Genomics, Chicago, USA) and manually corrected by comparison to the Swiss-Prot and TrEMBL databases [20], the analysis of functional domains with InterPro-Scan [21] and the use of the IMG/ER (Integrated Microbial Genomes/Expert Review) system [22].

### Sequence Analysis and Comparative Genomics

Deduced gene products were classified into functional categories performing a BLAST search against the COG database [23]. Comparative analyses of different Firmicutes was done as described previously [24] using a bidirectional BLAST algorithm combined with a global sequence alignment based on the Needleman-Wunsch algorithm [25]. ORFs were defined as orthologs at a similarity higher than 30% and a BLAST e-value lower than 10e-21. Visualizations of chromosome, plasmid and other DNA sequences were done by using DNAPlotter [26]. To identify metabolic pathways the pathway tools software from the BioCyc database collection [27] was employed. Reconstruction of pathways was manually curated. Alien genes and genomic islands were detected employing COLOMBO [28] and IslandViewer [29]. The 16S rRNA gene sequence-based calculations of phylogenetic affiliations were done employing the ARB software package [30].

### Semi-quantitative and End-point Reverse Transcriptase PCR (RT-PCR)

To analyze the transcription level of different genes total RNA was isolated from *C. acidurici* 9a cells with the QIAGEN RNeasy Mini kit (QIAGEN, Hilden, Germany) according to the manual of the manufacturer. Cells were grown in 20 to 30 ml medium with different uric acid concentrations and in the absence or presence of glycine. Initially, cells were sampled after 1 h of incubation at 37°C. Subsequently, samples were taken every 2 h until cells reached stationary growth phase (usually, after 9 h). After RNA isolation, remaining DNA was digested with the Ambion® TURBO DNA-*free*™ DNase (Ambion, Life Technologies GmbH, Darmstadt, Germany) according to the protocol of the manufacturer. The DNA digestion was evaluated by PCR with RNA as template and oligonucleotides specific for the constitutively expressed RNA polymerase gene (subunit A, *rpoA*). A standard PCR reaction was set up using the BIO-X-ACT™ short DNA polymerase (0.04 units/µl), OptiBuffer and MgCl_2_ according to the manufacturer’s guidelines (all Bioline, Luckenwalde, Germany). The PCR reactions were initiated at 98°C (2 min), followed by 30 cycles of 96°C (20 s), 60°C (20 s), 68°C (2 min), and ended with incubation at 72°C for 10 min. Genomic DNA of *C. acidurici* 9a served as positive control and nuclease-free water as negative control. RNA was quantified using a NanoDrop ND-1000 spectrophotometer (Peqlab Biotechnologie GmbH, Erlangen, Germany). Reverse transcription of the mRNA to cDNA was done employing the RevertAid™ H Minus First Strand cDNA synthesis kit (Fermentas, St. Leon-Rot, Germany) according to the instructions of the manufacturer. In each reaction, 5 µg of DNA-free RNA was used as starting material. Semi-quantitative PCR was carried out as described above, but the number of cycles was reduced to 20. The used oligonucleotides and the corresponding genes are listed in Table S1. To verify operon structures end-point RT-PCR was performed according to Passalacqua et al. [31].

### Plasmid Copy Number Determination

The plasmid copy number (PCN) was determined by the method of Škulj [32] with modifications. Cells of *C. acidiurici* 9a were harvested after 5 h exponential growth, frozen using liquid nitrogen, and stored at −80°C until use. After thawing, whole genomic DNA, including chromosomal and plasmid DNA, was isolated with the MasterPure™ Gram positive DNA purification kit (Epicentre Biotechnologies, Madison, USA) as recommended by the manufacturer. Oligonucleotides were deduced within the genes encoding the chromosomal DNA replication protein A (*dnaA*) and the plasmid replication protein B (*repB*) (Table S1). Serial dilutions were performed with the purified DNA as follows: undiluted, 1∶5, 1∶25, 1∶50, 1∶125 and 1∶250. Real-time PCR reactions (15 µl mixtures) containing Absolute Blue SYBR Green ROX mix (Fisher Scientific GmbH, Schwerte, Germany) were prepared as recommended by the manufacturer. Each reaction was performed in quadruplicate. Reactions were run on a Bio-Rad iQ5 real-time PCR detection system using the iQ5 optical system software version 2.1 (Bio-Rad Laboratories GmbH, München, Germany) for analysis. Cycling conditions were: 15 min at 95°C, followed by 40 cycles at 95°C (15 s) for denaturation, at 60°C (30 s) for annealing and at 72°C (30 s) for extension. A dissociation (melting) step at 95°C for 30 s was added, followed by 60°C (30 s) and 80 cycles ranging from 60 to 95°C (10 s) for melting. Threshold cycle (Ct) values were automatically generated by the iQ5 optical system software. The average Ct and standard deviation were calculated. Dilutions with standard deviation values above 0.3 were not used to determine amplification efficiency and copy number. Both were calculated according to Škulj [32]. For chromosome and plasmid different amplification efficiencies and Ct values were considered using the method of Pfaffl [33]. Calculations were carried out for each sample and dilution and the average and standard deviation were determined.

### Antimicrobial Resistance Tests

Stock solutions of antibiotics and antibacterial agents were prepared according to the guidelines of the manufacturers (AppliChem, Darmstadt, Germany and Sigma-Aldrich, Steinheim, Germany). We used the above-described standard liquid uric acid medium mixed with different concentrations of the respective antimicrobial agent [34]. The following 4 different concentrations of each tested drug were chosen and added to 10 ml of liquid uric acid medium: Ampicillin (20, 50, 100, 150 µg/ml), kanamycin (20, 50, 100, 150 µg/ml), chloramphenicol (5, 10, 30, 50 µg/ml), erythromycin (5, 10, 50, 100 µg/ml), vancomycin (5–40 µg/ml), spectinomycin (20, 50, 100, 200 µg/ml), gentamicin (10, 20, 50, 100 µg/ml), tetracycline (5, 10, 20, 30 µg/ml), bacitracin (10, 30, 50, 100 µg/ml), acriflavin (50, 150, 250, 350 µg/ml), 4-azaleucin (30, 50, 100, 200 µg/ml), thiamphenicol (2, 5, 15, 30 µg/ml), and clarithromycin (2, 5, 10, 20 µg/ml). The OD_600 nm_ was measured after 6 h and 24 h and compared to growth in uric acid medium without antibiotics. Tests were carried out in triplicate.

### Availability

The complete genome sequence of *Clostridium acidurici* 9a has been deposited in GenBank under accession numbers CP003326 (chromosome) and CP003327 (plasmid).

## Results and Discussion

### General Genome Features

**Figure 1:**
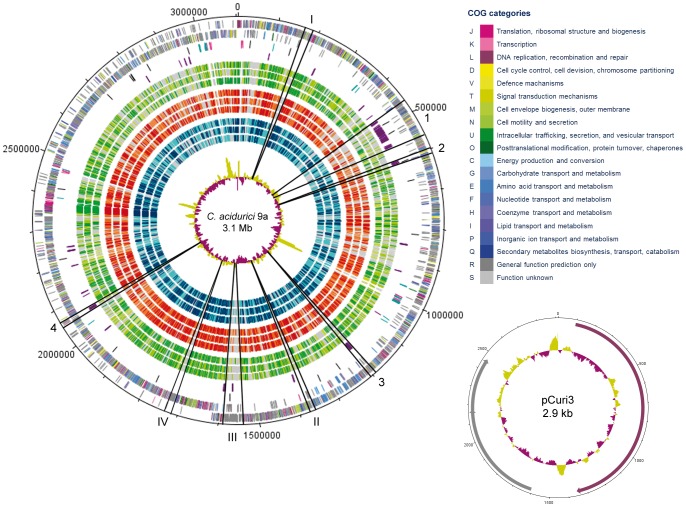
Circular maps of *C. acidurici* 9a chromosome and plasmid. Rings from the outside to the inside on the chromosome: 1 and 2, leading and lagging strand open reading frames (ORFs) colored according to COG categories; 3, rRNA cluster (pink), tRNAs (turquoise) and transposases (black); 4, predicted alien genes (purple); and 5–13, orthologous ORFs predicted with the Needleman-Wunsch algorithm. Organisms are roughly grouped into special substrates utilizers (green tones), solvent producers (red tones) and pathogens (blue tones). The shade of the corresponding color represents the value of the algorithm. Darker colors indicate higher values: dark green/red/blue, e-90/100; light green/red/blue, e-50/70: and grey, e-20/30, indicating no ortholog in the respective organism. Reference organisms from the outside to the inside: *Clostridium sticklandii* DSM 519, *Clostridium kluyveri* DSM 555, *Alkaliphilus oremlandii* OhILAs, *Clostridium ljungdahlii* DSM 13528, *Clostridium acetobutylicum* ATCC 824, *Clostridium beijerinckii* DSM 791, *Clostridium difficile* ATCC 9689, *Clostridium botulinum* A ATCC 25763 and *Clostridium tetani* E88. 14, G+C content within the chromosome below the average (magenta) and above the average (olive). Predicted genomic islands (arabic numerals) and unique regions (roman numerals) are separated by black lines. Rings from the outside to the inside on the plasmid: 1, open reading frames colored according to COG categories; and 2, G+C content below the average (magenta) and above the average (olive).

**Figure 2:**
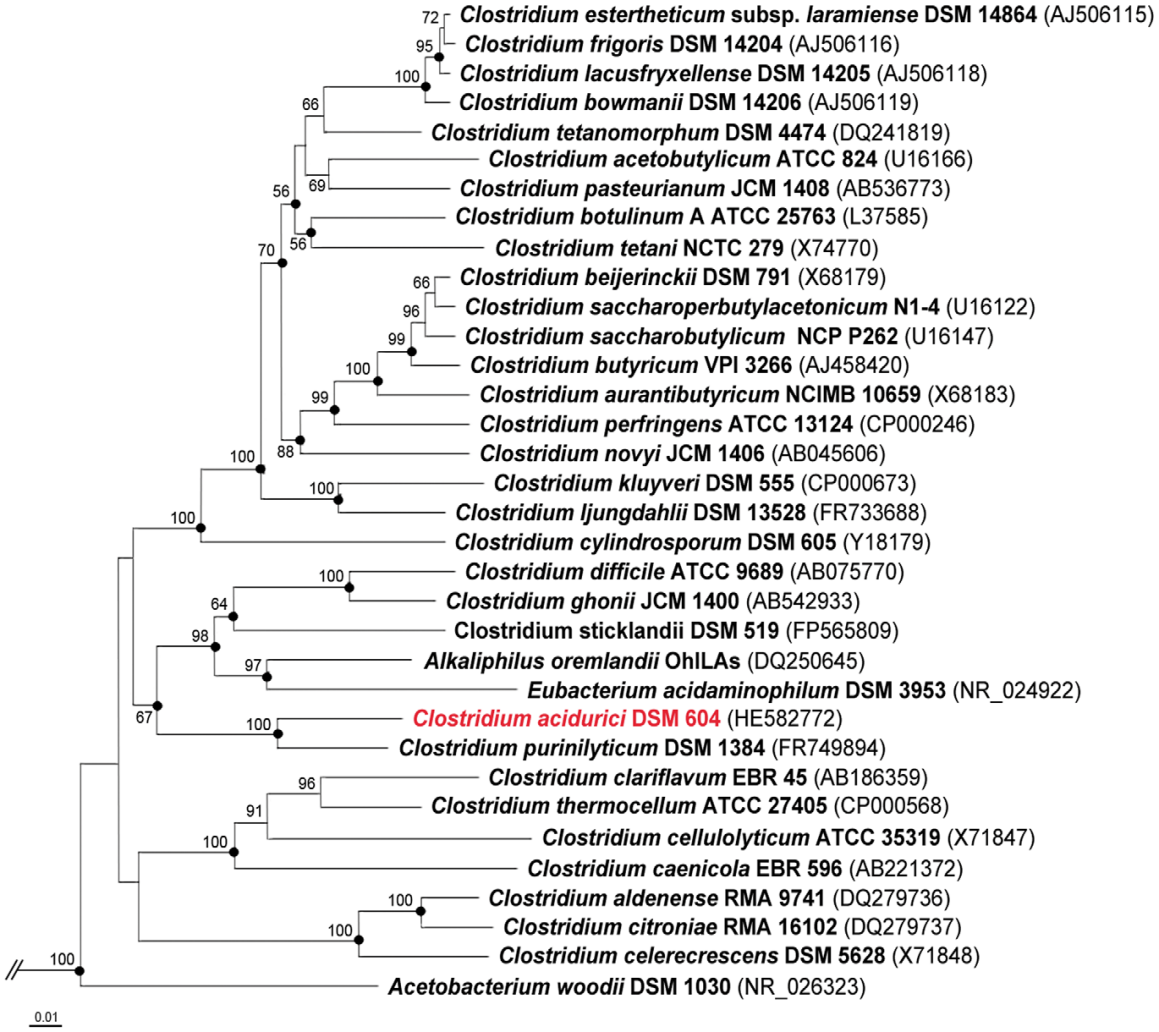
Taxonomic affiliation of *C. acidurici* 9a. The 16S rRNA gene sequences of type strains were used for construction of the neighbor-joining tree. GenBank accession numbers are given in parentheses. Numbers at nodes describe the bootstrap values in percent from 1,000 replicates. Bootstrap values >50% are shown. Black circles display reproducible nodes calculated with the maximum-likelyhood method. Length of the bar represents 0.01 substitutions per nucleotide position. *C. acidurici* 9a is marked in red. Tree calculation was done using the ARB software package [30]. The archaeal strains *Methanosarcina acetivorans* DSM 2834 (M59137), *Methanosarcina mazei* DSM 2053 (AJ012095) and *Methanosarcina barkeri* DSM 800 (AJ012094) were used as outgroups to define the tree root (not shown).

The genome of *C. acidurici* 9a consists of one chromosome with a size of 3,105,335 bp and a circular plasmid of 2,913 bp (Fig. 1). The G+C content of *C. acidurici* 9a (29.9%) is in the typical range (26–39%) known from other clostridia. Six rRNA clusters plus two additional single 5S rRNA genes and 81 tRNAs, including those responsible for selenocysteine synthesis, were identified in the genome of *C. acidurici* 9a (Fig. 1). The genome harbors 2979 predicted open reading frames (ORFs) of which 2878 are putative protein-encoding genes and 17 putative pseudogenes. A 16S rRNA gene-based phylogenetic classification (Fig. 2) confirmed the findings of Collins et al. [35] that *C. purinilyticum* was the closest relative of *C. acidurici* 9a. *C. cylindrosporum*, the third known purinolytic *Clostridium*, showed only a distant relationship to *C. acidurici* 9a. Thus, despite strong phenotypical similarities, a significant genotypic difference between *C. cylindrosporum* and *C. acidurici* 9a is indicated [34]. As an endospore-forming bacterium, *C. acidurici* 9a is equipped with a set of sporulation genes including universal transcription initiation factor *spo0A* (Curi_c13920), *spoIIAA-spoIIAB-sigF* operon (Curi_c10290-10310) (Fig. S1), *spoIIGA-sigE* operon (Curi_c13080-Curi_c13090) (Fig. S1), and the three sigma factors *sigG* (Curi_c13100), *sigK* (Curi_c13480) and *sigH* (Curi_c22810). As found in most other clostridia [36], the genome of *C. acidurici* 9a does not harbor genes encoding the phosphorelay components Spo0F and Spo0B. Recently, Steiner et al. discovered five orphan kinases in *C. acetobutylicum* that are able to overtake their function by interacting directly with Spo0A and controlling its phosphorylation [36]. We identified nine genes encoding putative orphan kinases in *C. acidurici* 9a of which three (Curi_c11130, Curi_c26240, Curi_c26310) show high similarities to the orphan kinases of *C. acetobutylicum*. *C. acidurici* 9a is motile and possesses two large cluster regions containing flagellar biosynthesis genes (Curi_c15800-16070 and Curi_c02130-02580). As described for *Clostridium ljungdahlii* [37], a cluster of chemotaxis genes (Curi_c15760-15790) is located directly in the neighborhood of the flagella gene clusters.

COG classification of protein-encoding genes revealed that many *C. acidurici* 9a genes are related to transcription (8.3%), amino acid transport and metabolism (8.4%), and signal transduction mechanisms (8.1%) (Fig. 1). A comparison of COG categories with 15 other *Clostridiaceae* (Table S2) showed lower representation of genes coding for carbohydrate transport and metabolism in *C. acidurici* 9a. Only 3.1% of all putative genes code for proteins of this category, whereas other clostridia show an average of 7.4%. This difference might be due to the narrow substrate spectrum of *C. acidurici* 9a.

### Alien Genes and Genomic Islands

Approximately 91 kb (2.9%) of the genome represent putative alien genes. Clusters of alien genes were located in 4 regions (Fig. 1). Most genes in *C. acidurici* 9a potentially derived from other bacteria are related to ABC transport systems such as Curi_c04900-04930 and Curi_c04500-04520, which encode a potential ferrichrome ABC-transporter and a transporter with unknown function, respectively. In region 1, harboring the ferrichrome ABC-transporter genes, putative alien genes mediating vancomycin resistance (Curi_c05040-05080) were detected. In addition, alien genes present within regions 1, 2, 3, and 4 code for acetyltransferases (Curi_c05130, Curi_c05320) and transcriptional regulators (Curi_c04890, Curi_c05120) or two-component signal transduction systems (Curi_c04810-04820, Curi_c05350-05360, Curi_c05440-05450). Since the genus *Clostridium* is heterogeneous and *C. acidurici* 9a employs a specialized way of life, we performed a bidirectional blast comparing the genome sequence of *C. acidurici* 9a to nine genome sequences of other clostridia and related bacteria to identify other traits or unique genes (Fig. 1). The organisms were divided into three subgroups based on the substrate spectra: (i) pathogens/proteolytic (*C. difficile* ATCC 9689, *C. botulinum* A ATCC 25763, *C. tetani* E88), (ii) solvent producer/saccharolytic/proteolytic (*C. beijerinckii* DSM 791, *C. acetobutylicum* ATCC 824, *C. ljungdahlii* DSM 13528), and (iii) utilizers of special substrates (*C. sticklandii* DSM 519, *A. oremlandii* OhILAs, *C. kluyveri* DSM 555). Four genomic regions of *C. acidurici* 9a showed low or no similarities to the other clostridia (Fig. 1, I–IV). These regions contained genes encoding S layer domain-containing proteins (I), uncharacterized acetyltransferases and transcriptional regulators (II) or hypothetical proteins (III and IV). The genome of *C. acidurici* 9a possesses 21 genes encoding transposases (Fig. 1) and genetic mobile elements, but some of these were identified as pseudogenes. No complete phages could be identified in the genome sequence but genes coding for remains of phages such as integrase family proteins (Curi_c05580, Curi_c24810, and Curi_c24830) and the small subunit of phage terminase (Curi_c14970) are present. In summary, the incorporation of foreign DNA into the genome by horizontal gene transfer is indicated.

### Purine Breakdown

**Figure 3:**
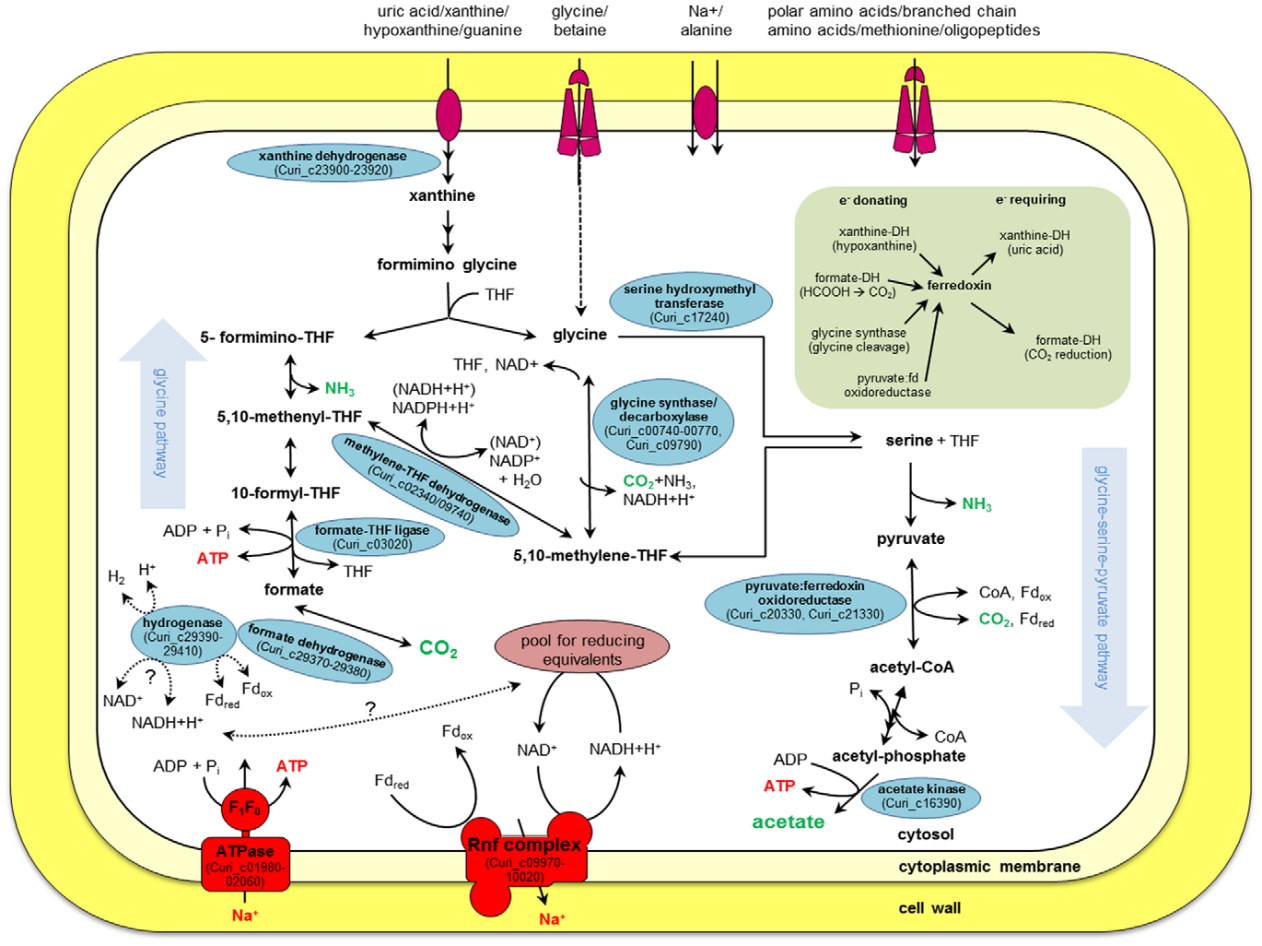
Metabolic pathways involed in purine utilization of *C. acidurici* 9a. The scheme represents a genome-based model of important metabolic and energy conservation steps during purine utilization of *C. acidurici* 9a. Relevant enzymes and their corresponding locus tags are shown in blue ellipses and end-products in green letters. Ferredoxin-involving steps (light green box) were modified after Vogels and van der Drift [53]. Abbreviations: Fd, ferredoxin; ox, oxidized; red, reduced; THF, tetrahydrofolate; ?, unknown.

Purines such as uric acid, xanthine, hypoxanthine and guanine serve as sole carbon, nitrogen and energy source of *C. acidurici* 9a, but adenine is not utilized [7], although putative genes coding for adenine deaminase (Curi_c04950, Curi_c09560, Curi_c29030) were detected in the genome. Products during growth on purines are acetate, CO_2_ and NH_3_ (Fig. 3). The first step in purine degradation is the conversion of the purine to xanthine. These reactions are mainly catalyzed by xanthine dehydrogenase. The xanthine dehydrogenase in *C. acidurici* 9a consists of three subunits, which bind molybdenum (XdhA), FAD (XdhB), or iron-sulfur (XdhC). We found one cluster of the corresponding genes in the genome (Curi_c23900-23920) and one additional single *xdhC* gene (Curi_c28980) that clusters together with genes coding for selenium-dependent molybdenum hydroxylase proteins (Curi_c28990-29010). A second cluster harbors an additional single *xdhA* gene (Curi_c19420) together with genes encoding the delta (Curi_c19430) and gamma subunit (Curi_c19440) of purine hydroxylase, which performs hydroxylation of hypoxanthine and purine to xanthine [38]. With guanine as substrate, guanine deaminase instead of xanthine dehydrogenase catalyzes the conversion to xanthine (Curi_c00270, Curi_c19450) [39]. When xanthine serves directly as substrate, 4-ureido-5-imidazole carboxylic acid is formed by the hydrolysis of the covalent bond between atoms 1 and 6 in the pyrimidine ring employing the enzyme xanthine amidohydrolase [40]. Although three putative genes coding for amidohydrolases (Curi_c02650, Curi_c26230, Curi_c27500) were identified in the genome, a specific one for xanthine could not be detected. Subsequently, 4-ureido-5-imidazole carboxylic acid is converted to formiminoglycine. The involved enzymes are characterized in *C. cylindrosporum* and partially in *C. acidurici* [40], [41]. However, the sequences of the corresponding genes are not published. Thus, we were not able to detect candidate genes encoding these enzymes in the genome of *C. acidurici* 9a. In addition, no gene encoding glycine formiminotransferse that converts formiminoglycine to glycine involving tetrahydrofolate (THF) was detected [40].

### Utilization of Glycine

#### Glycine as intermediate of purine degradation

The role of glycine as intermediate in purine fermentation and its degradation to acetate was studied in anaerobic acetogens and especially in the purinolytic bacteria *C. acidurici*, *C. cylindrosporum and C. purinilyticum* [4]–[6], [10]. Two pathways from glycine to acetate are known. The first one discovered was the glycine-serine-pyruvate pathway [42] (Fig. 3). Glycine is oxidized (decarboxylated) and the resulting methylene-THF condenses with another glycine to serine via serine hydroxymethyltransferase (GlyA, Curi_c17240). Serine is deaminated to pyruvate via L-serine dehydratase (SdhA/SdhB, Curi_c17690-17700), and pyruvate:ferredoxin oxidoreductase (Por, Curi_c20330, Curi_c21330) catalyzes the further conversion to acetyl-coenzyme A and CO_2_. A phosphotransacetylase (Curi_c12550) reversibly transfers the acetyl unit from CoA to phosphate and acetate is built from acetyl phosphate by the action of acetate kinase (Curi_c16390). The remaining CO_2_ can be partly reconverted to methylene-THF. An important element of this pathway is the glycine-cleavage system (termed also as glycine decarboxylase/synthase). As glycine decarboxylase, this multienzyme complex is responsible for the oxidation of glycine to methylene-THF, NAD(P)H, CO_2_ and NH_3_. The reverse glycine-forming reaction is also possible by the glycine synthase activity of the complex [9]. *C. acidurici* 9a possesses all genes necessary for encoding this enzyme complex, which includes genes encoding an aminomethyltransferase (*gcvT*, Curi_c00740), a heat-stable hydrogen carrier protein (*gcvH*, Curi_c00750), subunits of an α2/β2 tetrameric glycine dehydrogenase, which constitutes the decarboxylase in the system (*gcvPA* and *gcvPB*, Curi_c00760-00770), and the separately located dihydrolipoamide dehydrogenase (*lpd*, Curi_c09790). The conversion of formiminoglycine and THF to glycine also yields 5-formimino-THF, which is converted via different intermediates to formate by formate-THF ligase and further to CO_2_ by formate dehydrogenase. In the inverse direction (glycine pathway), acetate can be synthesized from CO_2_ via formate, serine, and glycine also involving the glycine cleavage system, now acting as glycine synthase (Fig. 3). In the overall reaction from glycine via serine and pyruvate to acetate, 4 glycine are converted to 3 acetate, 2 CO_2_ and 4 NH_3_. Additionally, 1 ATP is formed. For *C. purinilyticum* and *C. cylindrosporum* it has been shown that in the presence of selenite the energetically more favored glycine reductase pathway is pursued instead of the glycine-serine-pyruvate pathway [4], [5]. Glycine is reduced directly to acetate by a selenium-dependent glycine reductase, yielding 4 ATP. The required reduction equivalents are partly derived from total oxidation of 1 glycine via the glycine-cleavage system. Glycine reductase activity has been detected in *C. cylindrosporum* and *C. purinilyticum* [4]. In these organisms, the energetically less favorable glycine-serine-pyruvate pathway serves as an emergency pathway in the case of selenium deficiency [5]. It was always assumed that *C. acidurici* 9a is acting in the same way. However, Dürre and Andreesen [4] could not detect glycine reductase activity in this organism. They also performed labeling experiments with radioactive [2-^14^C]glycine and the labeling pattern for *C. acidurici* 9a differed from that of *C. purinilyticum* and *C. cylindrosporum*. Although ^14^C recovery was 30% lower and the conversion to [1-^14^C]acetate 15% lower than that of the other tested clostridia, the labeling pattern indicated the use of the glycine reductase pathway. However, the lack of potential genes coding for glycine reductase or subunits of this enzyme complex in the genome indicated the absence of the glycine reductase pathway in *C. acidurici* 9a. Genes encoding thioredoxin, which is the electron donor for the glycine reductase reaction [9], were not identified either, but genes encoding a thioredoxin reductase (*trxB*, Curi_c10870) and a thioredoxin-like protein (Curi_c20110) that shares similarities to a thiol:disulfide interchange protein were found in the genome of *C. acidurici* 9a. As in other bacteria [43], this result indicated the presence of an alternative thioredoxin in *C. acidurici* 9a.

#### Glycine as substrate

**Figure 4:**
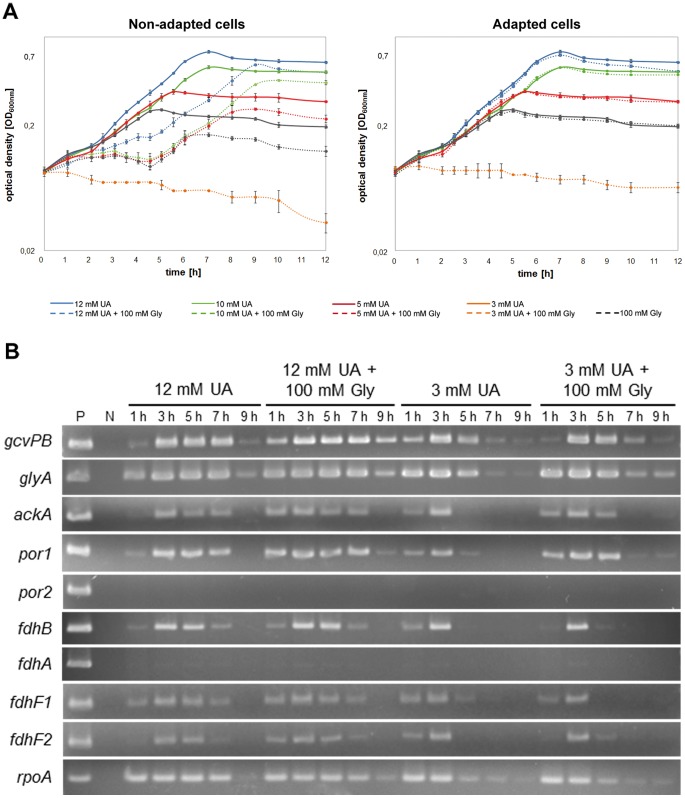
Growth on uric acid and glycine (A) and corresponding gene expression at different time points (B). (**A**) Growth of cells was measured over 12 h in medium containing different concentrations of uric acid (UA) with (dashed line) or without (solid line) glycine (Gly). Non-adapted cells were shifted directly from uric acid medium to uric acid with glycine whereas adapted cells were grown three times in 12 mM uric acid medium with 100 mM glycine prior to transfer and measurement. (**B**) Dependent on the addition of glycine a semi-quantitative analysis of transcription levels of genes involved in the conversion of glycine to acetate was performed. Shown are the results for adapted cells grown in 3 mM and 12 mM uric acid medium or in the same media supplemented with 100 mM glycine. Samples were taken after 1 h, 3 h, 5 h, 7 h and 9 h. DNA as template in the RT-PCR reaction served as positive control (P) and water as negative control (N). Abbreviations: *gcvPB*, glycine dehydrogenase beta subunit (Curi_c00770); *glyA*, serine hydroxymethyltransferase (Curi_c17240); *ackA,* acetate kinase (Curi_c16390); *por1/por2*, pyruvate:ferredoxin oxidoreductase (Curi_c20330/Curi_c21330); *fdhA*/*fdhB*, formate dehydrogenase subunits A; (Curi_c16670, Curi_c16640); *fdhF1/fdhF2*, formate dehydrogenase H (Curi_c29370/Curi_c29380); *rpoA*, DNA-directed RNA polymerase alpha subunit (Curi_c22350, reference).

Although glycine is an intermediate in the conversion of purines to acetate, it cannot be utilized as sole substrate by *C. acidurici* 9a [6]. Degradation and growth are only possible in the presence of a purine. The import of glycine into the cell might be facilitated by an ABC transport system. Three putative genes were identified, encoding a glycine/betaine ABC transporter (Curi_c10180-10200). To further analyze the glycine utilization of *C. acidurici* 9a, growth tests were performed in media containing glycine and different concentrations of uric acid. Tests were implemented with cells not adapted to glycine compared to adapted cells. Media containing glycine without uric acid served as negative control. As shown in Fig. 4A, non-adapted cells showed a longer lag phase than adapted cells when shifted from uric acid medium to glycine-containing medium. This was not surprising, as high concentrations of glycine exhibit an inhibitory effect on growth of many bacteria by disturbing the biosynthesis of peptidoglycan [9]. Adapted cells showed no extended lag phase and the maximal cell densities of 0.72, 0.57, 0.37, and 0.26 in glycine-containing medium with 12 mM, 10 mM, 5 mM, or 3 mM uric acid, respectively, were identical to those in medium containing solely uric acid. Growth of *C. acidurici* 9a depended only on the concentration of uric acid, since the addition of glycine showed neither an increase nor a decrease in optical density. To examine the influence of glycine on the conversion of glycine to acetate the transcription levels of genes involved in this process were determined. The selected genes code for the beta-subunit of the decarboxylase of the glycine cleavage system (*gcvPB*, Curi_c00770), serine hydroxymethyltransferase (*glyA,* Curi_c17240), pyruvate:ferredoxin oxidoreductase (*por,* Curi_c20330, Curi_c21330), acetate kinase (*ackA,* Curi_c16390), NAD^+^-dependent formate dehydrogenase (*fdhA*/*fdhB*, Curi_c16670/Curi_c16640) and formate dehydrogenases H (*fdhF1/fdhF2*, Curi_c29370-29380). Adapted cells were grown in media containing glycine and altering concentrations of uric acid and samples were taken in two hours intervals. In general, semi-quantitative RT-PCR revealed lower transcription levels of all tested genes when the uric acid concentration was decreased (Fig. 4B). The addition of glycine resulted in increased transcription levels of *gcvPB*, *glyA*, *ackA* and *por*, whereas the transcription of formate dehydrogenase genes slightly decreases. Although no transcription start analysis was performed, a potential glycine riboswitch with two aptamers (Curi_c00731) has been identified directly upstream of the genes encoding the glycine cleavage system. Thus, the increased gene expression might be due to the turn-on of the riboswitch [44]. The metabolism is forced to the direction of synthesizing C1 units and thereby increasing the transcription of *glyA*, *por* and *ackA*. We assume that in the presence of external glycine cells no longer need to synthesize it from purines and thus, less 5-formimino-THF is build. Accordingly, the formation of formate is reduced. This explains the decrease in transcription levels of genes encoding formate dehydrogenase. A second interesting aspect is the different transcription of *por2* and *fdhA.* In contrast to their orthologs, these two genes are not or only slightly transcribed. Nevertheless, no obvious sequence variations, which could explain the inactivity of the genes, were identified in their promoter regions. We assume these genes are not active under the conditions tested.

### Utilization of Sugars and Macromolecules

Sugars such as glucose were already tested as substrates in early studies by Barker and Beck [7]. Growth was analyzed in medium containing solely glucose as carbon source or glucose-containing medium supplemented with purines. Similar to the growth tests with glycine, growth was only observed after addition of an utilizable purine. In contrast to the studies with glycine, no decomposition of glucose could be detected [7]. In addition to glucose, we tested other hexoses and pentoses such as fructose, galactose, sucrose, ribose, and ribulose in media supplemented with different nitrogen sources such as ammonium chloride, ammonium sulfate, and ammonium nitrate, but growth was never observed (data not shown). This might be partly due to the fact that *C. acidurici* 9a does not possess a functional phosphotransferase system. We identified a putative EIIC component (Curi_c00230) and phosphocarrier protein Hpr (Curi_c20230) of a phosphotransferase system, but genes coding for unspecific enzyme I and other components of the substrate specific enzyme II are missing. Interestingly, all necessary genes coding for glycolysis/gluconeogenesis were identified in the genome of *C. acidurici* 9a, including genes encoding key enzymes such as phosphofructokinase (Curi_c20210) and pyruvate kinase (Curi_c20200). RT-PCR-based gene expression studies revealed that these genes are constitutively transcribed indicating that they have a metabolic function independent of sugar degradation. At least some of these enzymes are required for gluconeogenesis. Pyruvate kinase and phosphofructokinase may also be involved in purine or secondary metabolite metabolism. Additionally, putative genes involved in solvent production such as the genes encoded by the *sol* operon of *C. acetobutylicum* [45] were not identified in the genome sequence of *C. acidurici* 9a.

Except for a gene encoding a putative amylopullulanase (*apu*, Curi_c06010), putative genes coding for potential exoenzymes involved in degradation of macromolecules such as starch, proteins or lipids were not present. This again emphasizes the substrate specialization of *C. acidurici* 9a.

### Energy Conservation

**Figure 5:**
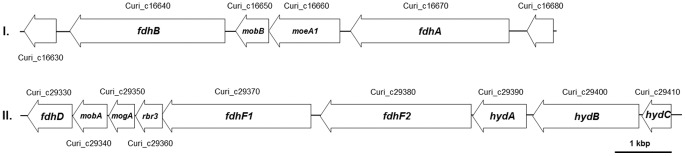
Organization and localization of the gene cluster encoding formate dehydrogenases. Cluster I, the genes *fdhA* and *fdhB* (Curi_c16640, Curi_c16670) encoding two subunits of NAD-dependent formate dehydrogenase are separated by two molybdopterin biosynthesis protein-encoding genes (Curi_c16650-16660); cluster II, genes *fdhF1* and *fdhF2* (Curi_c29370-29380) encoding formate dehydrogenase H cluster together with the genes *hydA*, *hydB* and *hydC* (Curi_c29390-29410) encoding three subunits of an iron-only hydrogenase. Downstream of this cluster, the formate dehydrogenase accessory protein encoding gene *fdhD* (Curi_c29330) is located.

Genes encoding two energy conservation steps during growth on purines were detected in the genome sequence. One is initiated by the hydrolysis of methenyl-THF to 10-formyl-THF followed by forming ATP and formate from ADP and 10-formyl-THF [46]. The second major energy-yielding step is the generation of acetate and ATP in the acetate kinase reaction (Curi_c16390). As described for other clostridia [37], [47], [48], *C. acidurici* 9a may also perform energy conservation by electron-transport phosphorylation employing the Rnf-complex (Curi_c09970-10020). This enzyme complex catalyzes the flow of electrons from reduced ferredoxin to NAD^+^ coupled to ion-translocation across the membrane. ATP is generated employing the F_1_F_0_-type ATPase (Curi_c01980-02060), which contains amino acid motifs typical for Na^+^-dependent F_1_F_0_ ATPases [49]. Recently, an ancient way of combining carbon dioxide fixation with the generation and utilization of a sodium ion gradient for ATP synthesis was described for *A. woodii* [50]. The Rnf-complex was shown to couple the electron transfer from reduced ferredoxin to NAD^+^ with electrogenic Na^+^ transport. The whole system plays an important role during growth on H_2_ and CO_2_ employing the Wood-Ljungdahl pathway [48]. For *C. acidurici* 9a and the other purinolytic clostridia, the glycine pathway was postulated and the necessary genes were identified in the genome of *C. acidurici* 9a. The glycine pathway shares the first steps with the Wood-Ljungdahl pathway [51]. The initial reduction of CO_2_ to formate, is catalyzed by the enzyme formate dehydrogenase. Most formate dehydrogenases are only able to function in the direction from formate to CO_2_, thus, the enzyme of acetogenic bacteria is rather unique being able to convert CO_2_ to formate [9]. As mentioned above, four genes encoding formate dehydrogenases were identified in the genome of *C. acidurici* 9a (Fig. 5). Two of them (*fdhB* and *fdhF2*) are coding for selenoproteins. The genes *fdhF1* and *fdhF2* encode formate dehydrogenase H. They are clustered together with three genes encoding proteins that show similarity to a NAD(P)^+^-dependent iron-only hydrogenase (*hydABC*, Curi_c29390-29410) that bears resemblance to the trimeric iron hydrogenase of *Thermotoga maritima* [52] and the HydABC subunits of the multimeric iron hydrogenase of *A. woodii* [50]. Accordingly, we assume a similar electron bifurcating mechanism. The formate dehydrogenase accessory protein-encoding gene *fdhD* (Curi_c29330) was identified downstream of this cluster. Two additional genes encoding putative hydrogenase HydA homologues were detected separated from the *hydABC* cluster. One (*hydA1*, Curi_c05020) is located in alien gene region 1 (Fig. 1) and the other one (*hydA2*, Curi_c05750) directly within a cluster of genes coding for proteins involved in cobalamin biosynthesis (Curi_c05740-05800). Interestingly, *C. acidurici* 9a was assumed to possess no or only low levels of hydrogenase activity [53]. The lack of H_2_ production has always been peculiar because *C. acidurici* 9a produces ferredoxin, which is mainly found in hydrogen-evolving species. The other two genes *fdhA* and *fdhB* encode catalytic subunits for the NAD^+^-dependent formate dehydrogenases. They are separated from each other by the genes *moeA* and *mobB* (Curi_c16650-16660), which are involved in molybdopterin biosynthesis (Fig. 5).

In the second step of the glycine pathway, formate-THF ligase (Curi_c03020) catalyzes the formation of formyl-THF from formate and THF under the consumption of one ATP. After this step the pathway of *C. acidurici* 9a diverts from that of most acetogens such as *A. woodii*. Via the conversion of several THF intermediates and the involvement of the glycine synthase system, glycine is formed and acetate is synthesized via serine and pyruvate [51]. In contrast to acetogens employing the Wood-Ljungdahl pathway, carbon monoxide dehydrogenase activity could not be measured in purinolytic acetogens [11]. Accordingly, only the putative gene encoding the subunit CooS of carbon monoxide dehydrogenase (Curi_c06440) has been detected in the genome of *C. acidurici* 9a.

### Intermediary Metabolism

Like most other anaerobes, *C. acidurici* 9a lacks an entire tricarboxylic acid (TCA) cycle but is able to pursue at least parts for biosynthetic purposes. A (Re)-citrate synthase in *C. acidurici* was already partially purified and described by Gottschalk in 1968 [54]. The coding gene (Curi_c11840) was identified in a cluster harboring also a gene encoding cis-aconitase (Curi_c11850), which converts citrate to cis-aconitate and isocitrate. The gene encoding isocitrate dehydrogenase (Curi_c28890) is located elsewhere and putatively encodes a NADP^+^-dependent enzyme. Glutamate can be synthesized directly from α-ketoglutarate by glutamate dehydrogenase (Curi_c02830) or by glutamate synthase via glutamine and glutamine synthetase (Curi_c11830). Fumarate is synthesized via aspartate employing aspartate aminotransferase (Curi_c15360, Curi_c23220), and argininosuccinate synthase and lyase (Curi_c0688-06890). Several clostridia produce high levels of corrins [55]. 5-Aminolevulinic acid is the general precursor for biosynthesis of corrins and other tetrapyrroles. Two pathways for the synthesis of 5-aminolevulinic acid are known. In *C. acidurici* 9a the whole set of genes for synthesis of 5-aminolevulinic acid from glutamate (C5 pathway) is present. The genes encoding the key enzymes glutamate-1-semialdehyde aminotransferase (*hemL,* Curi_c24630) and glutamyl-tRNA reductase (*hemA,* Curi_c24690), are clustered together with *hem*B, *hem*C and *hemD*, which are required to synthesize uroporphyrinogen-III from 5-aminolevulinic acid and corrins such as coenzyme B_12_ (Curi_c24680-24950). This gene cluster is interrupted by a cluster of genes encoding phage integrase family proteins and hypothetical proteins (Curi_c24760-24880). Genes for biosynthesis of heme were absent in the genome of *C. acidurici* 9a, indicating an inability to form cytochromes.

### Plasmid Characterization

The small circular plasmid of *C. acidurici* 9a (2,916 bp) harbors only three predicted ORFs (Fig. 1) of which one encodes a RepB family replication initiation protein (Curi_3p00010). The other two putative genes encode proteins of unknown function (Curi_c3p00020-00030). These so-called cryptic plasmids with no known or obvious function occur often within bacteria, especially in staphylococci [56]. Two examples for cryptic clostridial plasmids are pSMBb and pCB101 of *C. acetobutylicum* DSM 1731 and *C. butyricum* NCIB 7423, respectively [57]. The number of plasmid copies per chromosome (PCN) of *C. acidurici* 9a was estimated by quantitative real-time PCR in cells harvested in exponential phase. For this purpose, the occurrence on DNA level of a unique gene on the plasmid was used for comparison to a unique gene on the chromosome. We chose the genes for the plasmid replication initiation protein (Curi_3p00010) and the chromosomal replication protein DnaA (Curi_c00010). The observed PCN value of 9±1 for the *C. acidurici* 9a plasmid indicates a low-copy plasmid. Gene expression studies of both plasmid-derived genes coding for hypothetical proteins showed expression of both during exponential growth (Fig. S2). This indicated an important but so far unknown function of both hypothetical proteins. In addition, the plasmid remains stable even after more than 30 transfers.

### Antimicrobial Resistance

The *C. acidurici* 9a genome contains several genes similar to known genes mediating resistance against antimicrobials (Table S3). Responses to some antibiotics such as kanamycin and tetracycline were already tested by Schiefer-Ullrich et al. [34]. Since the reported results are partly inconsistent with the genomic findings, especially regarding resistance against bacitracin, a series of resistance tests was performed. *C. acidurici* 9a reacted sensitive towards kanamycin and tetracycline (Table S4). These findings are supported by the lack of corresponding resistance-encoding genes in the genome sequence. We also observed sensitivity against gentamicin, which belongs to the same group of antibiotics as kanamycin. Interestingly, these antibiotics are known to be most active against Gram-negative bacteria and are usually inactive against strict anaerobes, as transport of the aminoglycoside into cells is facilitated through an oxygen-consuming process [58]. Similar to Schiefer-Ullrich et al. [34] a slight resistance to the glycopeptide vancomycin, which inhibits peptidoglycan crosslinking, has been observed. In enterococci, six types of vancomycin resistances have been identified [59]. All function similar but possess a partly different structure. Two gene clusters encoding vancomycin resistance enzymes were identified in *C. acidurici* 9a (Table S3). One harbors the two-component signal transduction system VanR-VanS (Curi_c20130-20140 and genes encoding the D-alanyl-D-alanine carboxypeptidase VanY (Curi_c20120), which prevents vancomycin-binding to the peptidoglycan precursors. In the same region additional genes coding for VanY (Curi_c20120) and a VanZ-like protein (Curi_c20080) were identified. The second cluster contains genes (Curi_c05040-05080) coding for the vancomycin B/G-type resistance protein VanW, the D-alanine-D-serine ligase VanG, the D-alanyl-D-alanine carboxypeptidase VanXY and the serine-type alanine racemase VanT. A gene encoding a second putative VanR is also present, but the corresponding kinase VanS is missing. Taking the findings in *Entercoccus faecalis* [60] into account we postulate a G-type vancomycin resistance for *C. acidurici* 9a. Additionally, G-type resistance systems are known to mediate only moderate or rather weak resistance against vancomycin in enterococci (MIC = 16 µg/ml) [60]. Similar results were observed for *C. acidurici* 9a (MIC approximately 20 µg/ml, Table S4). However, in contrast to *E. faecalis*, the transcriptional regulation unit plus the additional VanY are separated from the resistance-mediating genes in *C. acidurici* 9a. Furthermore, G-type resistant *E. faecalis* possesses upstream of *vanR* a second gene encoding a vancomycin transcriptional regulator called VanU. This regulatory protein was absent in the *C. acidurici* 9a genome. Other tested antibiotics were ampicillin, bacitracin, chloramphenicol and erythromycin. Schiefer-Ullrich et al. observed sensitivity against these antibiotics [34]. In our tests, growth was observed in all cases (Table S4) and especially bacitracin showed almost no effect on growth of *C. acidurici* 9a, indicating a high resistance against this antibiotic. In accordance, *C. acidurici* 9a possesses a bacitracin ABC export system (Curi_c27110-27120) and an additional bacitracin resistance protein (Curi_c18810). Finally, putative genes encoding resistance proteins against acriflavine (Curi_c03050-03080, Curi_c29460-29480) and azaleucine (Curi_c06570, Curi_c25350-25360) were detected in the *C. acidurici 9a* genome. Susceptibility tests revealed that *C. acidurici* 9a possesses high tolerance against both compounds as no growth inhibition was visible at the highest tested concentrations.

**Figure 6:**
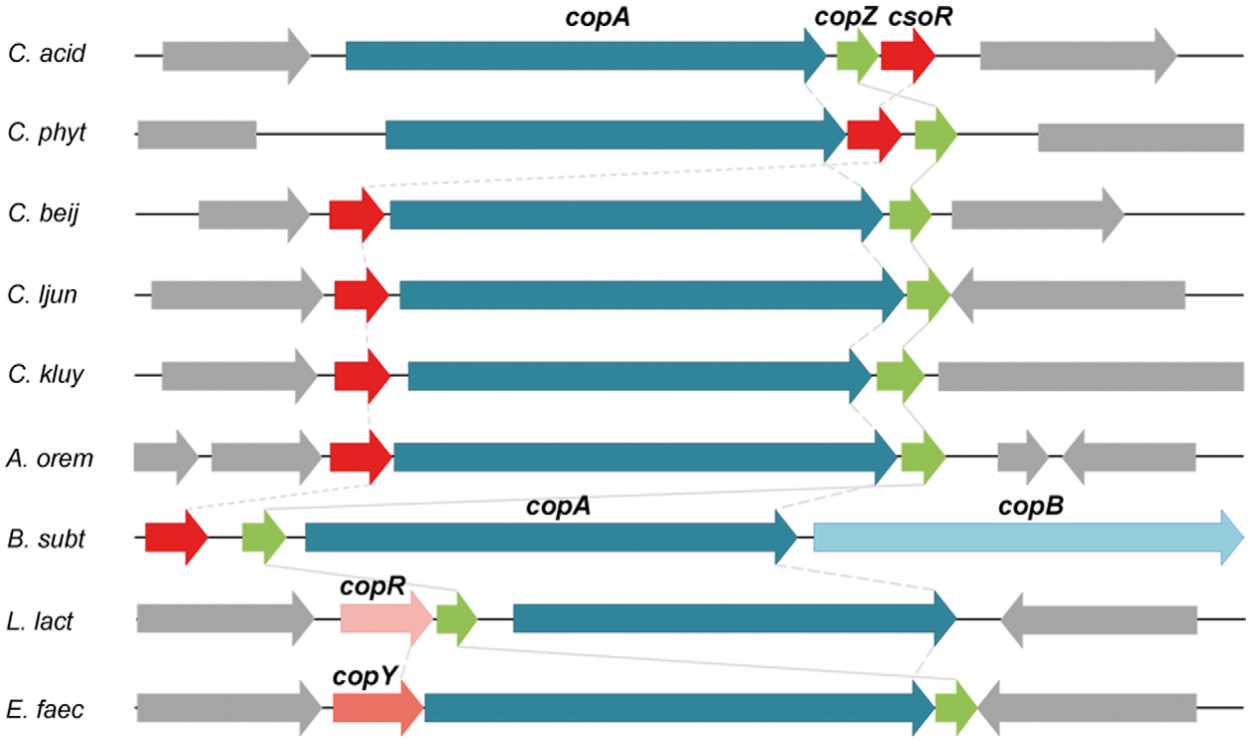
Organization of the *cop* operon in several bacteria. Organization of the *copAZ-csoR* locus of *C. acidurici* 9a (*C. acid*) compared to the copper homeostatic systems of *C. phytofermentans* (*C. phyt*), *C. beijerinckii* (*C. bei*), *C. ljungdahlii* (*C. ljun*), *C. kluyveri* (*C. kluy*), *Alkaliphilus oremlandii* (*A. orem*), *Bacillus subtilis* (*B. subt*), *Lactococcus lactis* (*L. lact*) and *Enterococcus faecalis* (*E. faec*.). Genes encoding the copper P-type ATPases *copA* and *copB* are shown in blue and light blue, respectively. Genes coding for the copper chaperone *copZ* are green and the different types of transcriptional repressors are depicted in red tones. Neighboring genes are shown in grey.

Additional to resistance genes against antibiotics, we also identified various genes that encode proteins similar to those involved in metal resistance in other organisms (Table S5). Besides chromate transport protein-encoding genes (Curi_c01850-01860, Curi_c04760) and one putative gene encoding an aluminium resistance protein (Curi_c15240), genes coding for a complete copper homeostasis system (Curi_c01370-01390) were identified. The copper homeostasis system-encoding genes are organized in an operon (*cop* operon, Fig. 6A, Fig. S1). It consists of a copper-translocating P-type ATPase (CopA, Curi_c01370), a copper chaperone (CopZ, Curi_c01380) and a copper-sensing transcriptional repressor (CsoR, Curi_c01390). In *Enterococcus faecalis* and *Lactococcus lactis*, the expression of the *cop* operon is modulated by the CopY-type transcriptional repressors CopY or CopR. *B. subtilis* and clostridia such as *C. acidurici* 9a employ CsoR-type transcriptional repressors. They are tetrameric and each homodimer binds two Cu^+^ forming a bridge between the subunits. This construct binds to a 30 bp-region upstream of the *cop* operon overlapping its promoter. Binding of additional Cu^+^ is thought to cause a conformational change of CsoR that leads to dissociation from the DNA [61]. In all shown organisms (Fig. 6) the transcriptional repressor-encoding gene is located upstream of the remaining *cop* operon genes whereas in *C. acidurici* 9a it is located downstream at the end of the operon. In *B. subtilis, csoR* is transcribed from its own promoter, as well as *copZA* [62]. This is different in *C. acidurici* 9a as a promoter search suggests a promoter region upstream of *copA* but not in front of *copZ* or *csoR* (Fig. S3).

We also identified two putative genes involved in cation detoxification. One encodes an ortholog of the P-type ATPase CadA (Curi_c06400), which mediates Cd, Co and Zn resistance. A putative gene encoding the corresponding transcriptional regulator CadC was not found. The other one encodes the Zn/H(+)-K(+) antiporter CzcD (Curi_c26810). First experiments indicated tolerance against copper and zinc (data not shown).

### Conclusions

In this study, we present the first complete genome sequence of a purinolytic *Clostridium.* The annotation of *C. acidurici* 9a enabled a reconstruction of its energy metabolism. Since the genome revealed the absence of genes encoding glycine reductase, its metabolism has to be reconsidered. Alternatively, we postulate the employment of the glycine-serine-pyruvate pathway for which all genes have been identified in the genome. Furthermore, our results show that additional glycine in the medium leads to a shift of expression of genes involved in acetate formation. We could confirm that in the presence of glycine expression levels of genes encoding the glycine cleavage system and conversion of glycine to acetate via serine and pyruvate increase. In contrast, genes encoding formate dehydrogenase are less transcribed indicating a reduced usage of the formate-CO_2_ branch.

Analysis of the genome sequence indicated the presence of resistance mechanisms against several antimicrobials such as bacitracin and acriflavine or copper and zinc, respectively. The cryptic plasmid pCuri3 of *C. acidurici* 9a is a low-copy plasmid. It merely encodes a replication protein and two hypothetical proteins with unknown function. Nevertheless, the hypothetical genes seem to mediate important functions, as they are constitutively transcribed and the plasmid is very stable. *C. acidurici* 9a possesses the full genetic equipment for glycolysis including the key enzymes, although it is not able to grow on hexoses and pentoses. This inability might be partly due to the absence of a functional phosphotransferase system. In the future, it would be interesting to test the ability to restore growth on sugars of *C. acidurici* 9a by transfer of genes encoding a complete phosphotransferase system. Possible candidates would be the fructose-specific or glucose-specific phosphotransferase systems of *C. acetobutylicum*. For this purpose, the establishment of a gene transfer method for *C. acidurici* 9a would be necessary. A recently developed system for constructing customized stable replicative shuttle plasmids, the pMTL80000 modular plasmids, could provide an effective way to genetically access *C. acidurici* 9a, as it was successfully employed for several other clostridia [63]. Alternatively, the cryptic plasmid pCuri3 could be modified to serve as shuttle vector.

## Supporting Information

Figure S1 **Operon structure verification with end-point RT-PCR.**
(PDF)

Figure S2 **Semi-quantitative transcription analysis of plasmid-related genes.**
(PDF)

Figure S3 **Schematic depiction of the** ***copAZ-csoR*** **locus and its estimated promoter region.**
(PDF)

Table S1 **Oligonucleotides used for RT-PCR and Real-time qPCR.**
(PDF)

Table S2 **COG categories of the predicted genes encoded by the** ***C. acidurici*** **9a genome and genomes of other selected clostridia.**
(PDF)

Table S3 **Genes encoding antibiotic resistance proteins of** ***C. acidurici*** **9a.**
(PDF)

Table S4 **Antibiogram of** ***C. acidurici*** **9a.**
(PDF)

Table S5 **Genes encoding metal resistance proteins of** ***C. acidurici*** **9a.**
(PDF)
