# Supplementary material for: The Purine-Utilizing Bacterium Clostridium acidurici 9a: A Genome-Guided Metabolic Reconsideration
Source: PLoS One. 2012 Dec 11;7(12):e51662. doi: 10.1371/journal.pone.0051662 (PMC3519856; doi:10.1371/journal.pone.0051662)
Supplement: Figure S1 — Operon structure verification with end-point RT-PCR. (PDF) [file pone.0051662.s001.pdf]

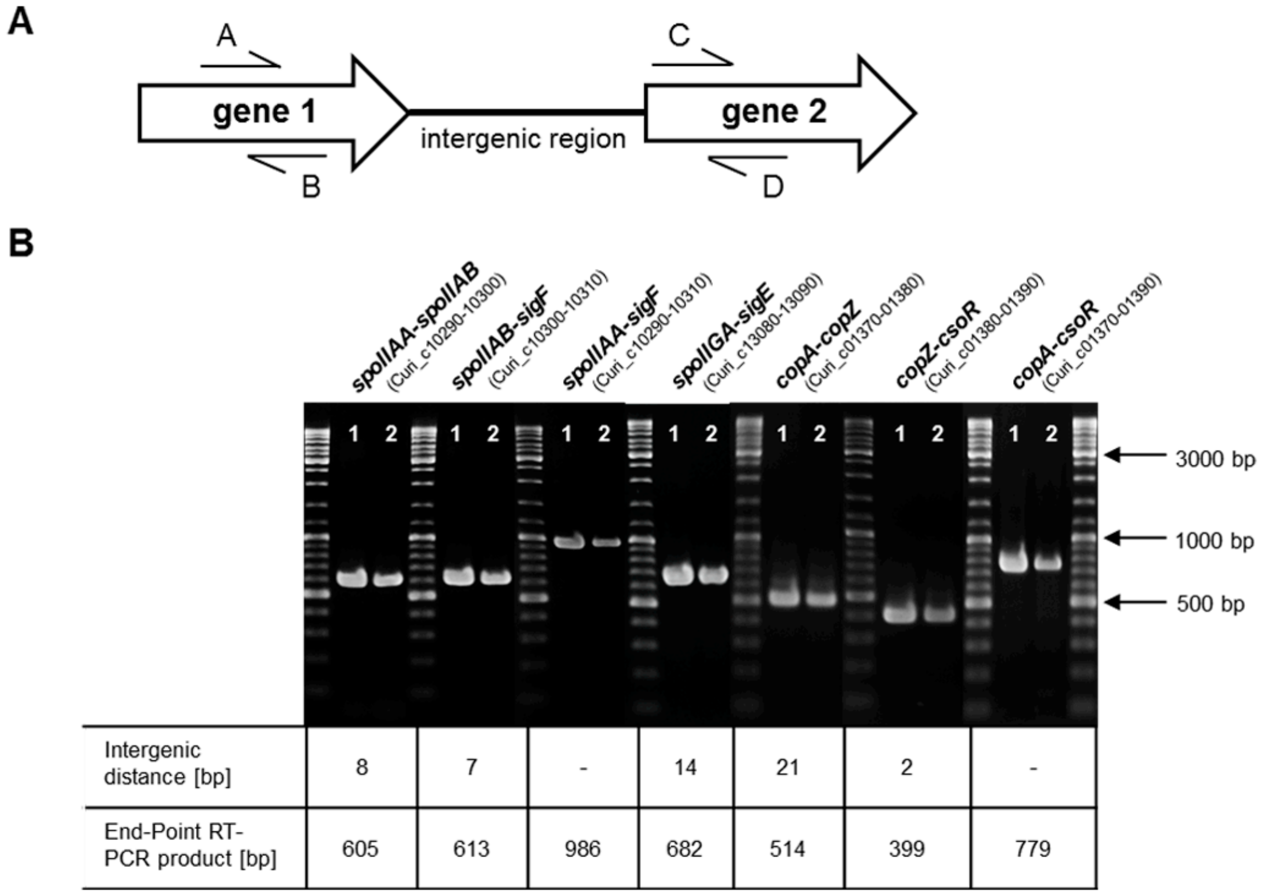

Intergenic distance [bp]	8	7	-	14	21	2	-
End-Point RT-PCR product [bp]	605	613	986	682	514	399	779

Figure S1. Operon structure verification with end-point RT-PCR. (A) Schematic method overview of End-Point RT-PCR (Passalacqua et al. 2009). Oligonucleotides A, B, C, and D were designed to amplify products within the genes (A+B and C+D) or, in the case of a contiguous mRNA transcript, across the intragenic regions (A+D). **(B)** Agarose gel electrophoresis of co-operonic gene pairs using oligonucleotides A and D and corresponding sizes of the End-Point RT-PCR products and intergenic distances. Operon structures that were verified included the sporulation operons *spoIIAA-spoIIAB-sigF* (Curi_c10290-10310) and *spoIIGA-sigE* (Curi_c13080-13909), and the copper homeostasis operon *copA-copZ-csoR* (Curi_c01370-01390). Cells were harvested after 5 h within the exponential phase. Whole RNA was isolated and end-point RT-PCR was performed on cDNA with 30 cycles of amplification (2). PCR reactions with oligonucleotides A and D on genomic DNA served as positive control (1). Intragene products with oligonucleotides A+B and C+D and PCRs with water or RNA as template served as further controls (not shown).

Reference

Passalacqua KD, Varadarajan A, Ondov BD, Okou DT, Zwick ME, et al. (2009) Structure and complexity of a bacterial transcriptome. J Bacteriol 191: 3203-3211.
