# Supplementary material for: The Purine-Utilizing Bacterium Clostridium acidurici 9a: A Genome-Guided Metabolic Reconsideration
Source: PLoS One. 2012 Dec 11;7(12):e51662. doi: 10.1371/journal.pone.0051662 (PMC3519856; doi:10.1371/journal.pone.0051662)
Supplement: Figure S2 — Semi-quantitative transcription analysis of plasmid-related genes. (PDF) [file pone.0051662.s002.pdf]

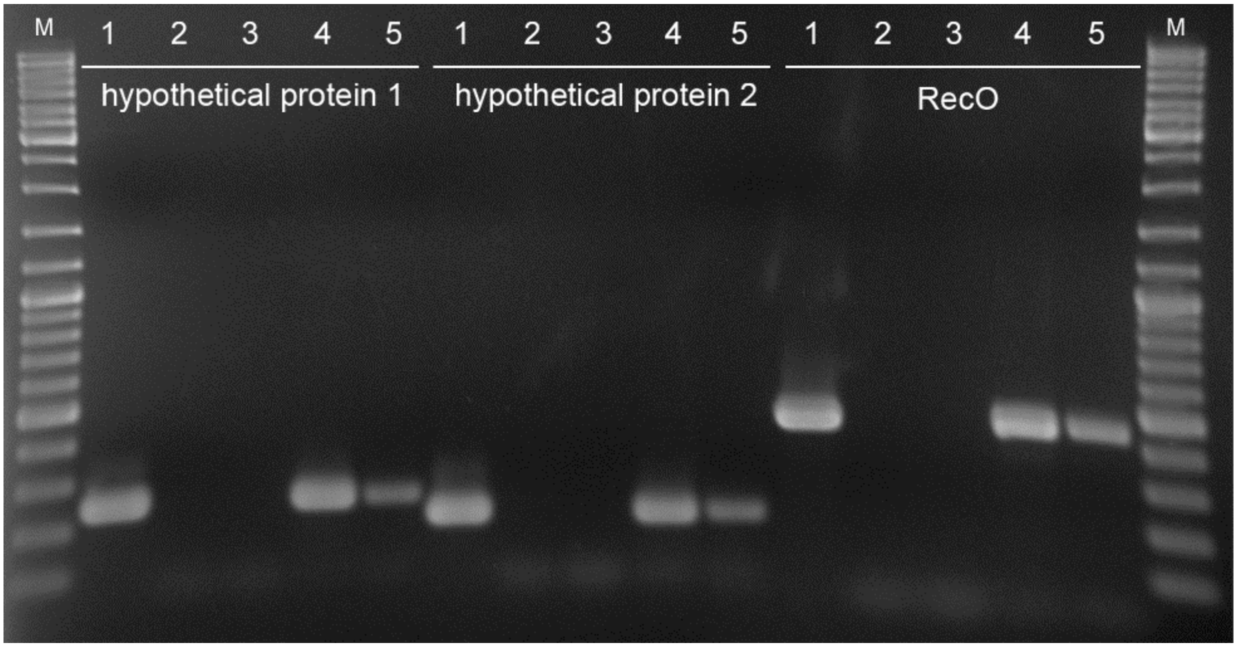

Figure S2. Semi-quantitative transcription analysis of plasmid-related genes. Cells were harvested within the exponential phase (after 5 h). Whole RNA was isolated and RT-PCR was performed to analyze the expression with primers specific for the two genes present on the cryptic plasmid pCuri3 encoding two hypothetical proteins (hypothetical protein 1, Curi_3p00020; hypothetical protein 2, Curi_3p00030). The gene *recO* (repair protein RecO, Curi_c18090) was used as constitutively transcribed positive reference. DNA as template served as positive control (1) and water (2) and RNA (3) as negative controls, respectively. RT-PCR was done using cDNA with 30 cycles of amplification (4) and 20 cycles (5) in comparison for a semi-quantitative analysis. The Gene Ruler ladder mix (Fermentas, St. Leon-Rot, Germany) served as standard DNA marker (M).
