# Supplementary material for: The Purine-Utilizing Bacterium Clostridium acidurici 9a: A Genome-Guided Metabolic Reconsideration
Source: PLoS One. 2012 Dec 11;7(12):e51662. doi: 10.1371/journal.pone.0051662 (PMC3519856; doi:10.1371/journal.pone.0051662)
Supplement: Figure S3 — Schematic depiction of the copAZ-csoR locus and its estimated promoter region. (PDF) [file pone.0051662.s003.pdf]

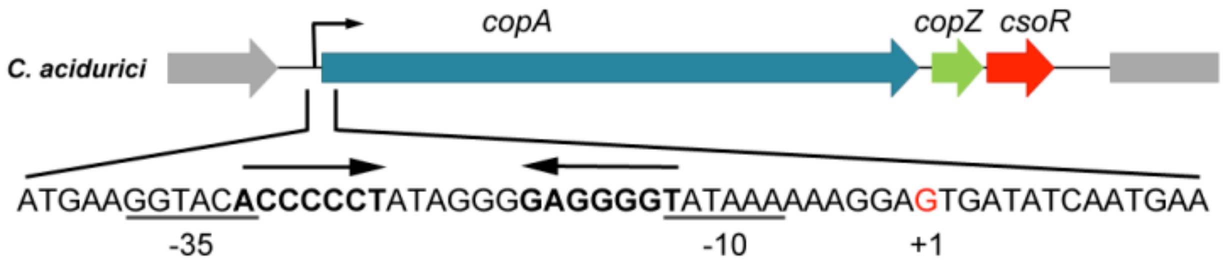

Figure S3. Schematic depiction of the *copAZ-csoR* locus and its estimated promoter region. The promoter region of the *copAZ-csoR* operon in *C. acidurici* 9a has been expanded to illustrate the different estimated promoter elements: -35 and -10 regions are underlined, +1 site is colored in red and bold letters represent the assumed GC-rich pseudo-inverted repeat serving as binding motif for CsoR.
