# Supplementary material for: The Purine-Utilizing Bacterium Clostridium acidurici 9a: A Genome-Guided Metabolic Reconsideration
Source: PLoS One. 2012 Dec 11;7(12):e51662. doi: 10.1371/journal.pone.0051662 (PMC3519856; doi:10.1371/journal.pone.0051662)
Supplement: Table S1 — Oligonucleotides used for RT-PCR and Real-time qPCR. (PDF) [file pone.0051662.s004.pdf]

Table S1. Oligonucleotides used for semi-quantitative RT-PCR and Real-time qPCR

**Application**	**Primer**	**Target Gene**	**Size**	**Sequence (5'-3')**	T_{PCR}
**Real-Time qPCR**	rep_for	*repB*	215 bp	TAA CTC AAG CTG TCC GAA TGG	60 °C
	rep_rev			CAC CTT CAT ATC CCC AGG AG	59 °C
	dna_for	*dnaA*	184 bp	GAG GAT AGA CTG CGT TCG AGA	60 °C
	dna_rev			AGT GCT CCT TCT AGT TCC CTG ATA TT	61 °C
**RT-PCR**	rpo_for	*rpoA*	126 bp	TTC TCT CAG CTG GCA CAT AGC	61 °C
	rpo_rev			ATT GAT ACG GGT TCG GAA GTG	61 °C
	gcv_for	*gcvPB*	429 bp	TGA AAT AGC AGG TTT CGC AAG	60 °C
	gcv_rev			TAC GTC AAA TCC CAT GTC TCC	60 °C
	por1_for	*por1*	342 bp	ACA CTA CCT GAC GCA AGC ATT	60 °C
	por1_rev			CAT CTT CGA CTA TGG CAG AGC	60 °C
	por2_for	*por2*	267 bp	GAT GAA CAA CCA GTC GCA TTT	60 °C
	por2_rev			TAT GTC CAG CAC CAG AAA AGG	60 °C
	fdhB_for	*fdhB* seleno	375 bp	TAT TTT GTC CAC GCA TTG GAT	60 °C
	fdhB_rev			GCC ACA CTA TGG CTT CCA TTA	60 °C
	fdhA_for	*fdhA*	414 bp	CCT ATG ATT GGA TGG GCT TCT	60 °C
	fdhA_rev			GCG ATG TGT GTT AAA GGG AGA	60 °C
	fdhF1_for	*fdhF1*	267 bp	TTG GTT CCT GAT GTG TGT TGA	60 °C
	fdhF1_rev			TAA GCC GGG AAC GAA TAT AGC	60 °C
	fdhF2_for	*fdhF2* seleno	322 bp	AGC TGG TAA AAC GAC ATC TGC	59 °C
	fdhF2_rev			ATG TAC AGG GAG CTT GCG ATA	60 °C
	gly_for	*glyA*	292 bp	ACG AAG TCA GCG TGT GGT ACT	60 °C
	gly_rev			TTA ACT CAC GGA AGC CCT GTA	60 °C
	ack_for	*ackA*	210 bp	CAT TGT TTG ATG GAA CGC TGT	61 °C
	ack_rev			TGA TGC GGT AGG TCA TAG AGT TG	61 °C

$\mathbf{T_{PCR}}$, used annealing temperature in PCR reaction
