# Supplementary material for: The Purine-Utilizing Bacterium Clostridium acidurici 9a: A Genome-Guided Metabolic Reconsideration
Source: PLoS One. 2012 Dec 11;7(12):e51662. doi: 10.1371/journal.pone.0051662 (PMC3519856; doi:10.1371/journal.pone.0051662)
Supplement: Table S2 — COG categories of the predicted genes encoded by the C. acidurici 9a genome and genomes of other selected clostridia. (PDF) [file pone.0051662.s005.pdf]

Table S2. COG categories of the predicted genes encoded by the *C. acidurici* 9a genome and genomes of other selected clostridia.

	Genes in COG categories (%)																			
	**E**	**G**	**D**	**N**	**M**	**H**	**V**	**C**	**S**	**R**	**P**	**U**	**I**	**F**	**O**	**L**	**Q**	**T**	**K**	**J**
***Clostridium acidurici* 9a**	8.4	3.1	1.3	3.4	4.5	5.4	2.9	4.7	9.5	10.3	6.9	2.1	1.9	2.6	3.2	5.3	1.0	8.1	8.3	6.5
***Clostridium acetobutylicum* ATCC 824**	7.4	8.3	1.2	3.9	6.8	4.4	2.8	4.4	9.0	11.7	3.9	1.6	2.5	2.5	2.9	4.5	1.1	6.8	8.9	5.4
***Clostridium bartlettii* DSM 16795**	10.6	6.2	1.1	0.2	4.5	4.5	3.5	5.7	8.4	13.4	6.2	1.0	1.6	3.3	3.2	5.2	0.9	4.9	8.5	4.9
***Clostridium beijerinckii* NCIMB 8052**	7.2	11.5	1.0	3.1	4.6	3.6	2.1	6.2	7.7	11.8	4.0	1.1	2.4	2.1	2.7	3.9	1.3	8.6	11.2	4.1
***Clostridium botulinum* F Langeland**	8.7	4.7	1.2	2.7	5.4	4.1	3.1	6.5	8.8	11.8	5.2	1.4	2.1	2.8	2.8	4.8	1.0	6.8	10.1	6.1
***Clostridium difficile* 630**	8.9	9.1	0.9	1.8	5.2	3.3	2.8	6.1	7.7	10.4	4.7	1.8	1.7	2.7	2.3	5.1	1.1	7.8	11.7	4.9
***Clostridium ljungdahlii* DSM 13528**	9.9	5.1	1.1	3.1	5.9	5.1	2.7	7.0	7.5	10.9	4.6	1.3	1.8	2.4	2.5	4.2	1.5	8.4	10.2	4.7
***Clostridium hiranonis* DSM 13275**	8.2	5.6	1.1	0.3	6.4	4.1	2.7	6.4	8.0	12.7	4.4	1.2	2.3	3.9	3.4	5.9	1.4	5.2	9.0	8.0
***Clostridium hylemonae* DSM 15053**	9.2	12.9	1.2	1.4	4.6	3.6	3.0	6.5	6.6	11.6	3.6	1.2	2.1	2.9	2.4	3.7	1.4	7.1	10.1	5.2
***Clostridium kluyveri* DSM 555**	8.7	4.2	1.1	3.1	5.4	4.5	2.9	6.8	8.4	10.4	5.1	1.9	2.2	2.2	3.3	6.4	1.8	7.1	5.0	5.2
***Clostridium novyi* NT**	8.0	5.8	1.7	3.5	6.2	5.0	2.4	5.8	7.8	10.7	4.2	2.1	2.6	3.2	3.5	6.7	0.7	5.9	6.4	8.0
***Clostridium phytofermentans* ISDg**	7.6	11.8	1.2	2.5	4.7	3.0	3.4	6.0	7.6	11.4	4.5	1.3	1.8	2.5	3.2	4.3	1.2	7.0	9.5	5.5
***Clostridium saccharolyticum* DSM 2544**	8.5	13.7	1.0	1.8	4.7	3.1	2.1	5.3	6.7	11.7	4.6	1.5	1.7	3.0	2.6	5.7	1.1	6.8	9.7	4.9
***Clostridium sticklandii* DSM 519**	10.2	4.2	1.6	3.6	4.4	5.1	1.9	6.7	9.2	11.1	5.2	2.0	2.1	2.7	2.9	5.3	1.2	7.1	6.5	7.0
***Clostridium tetani* E88**	8.2	4.5	1.3	3.4	6.5	4.4	2.4	6.3	7.9	11.6	5.3	1.7	2.7	3.0	2.9	6.8	0.9	6.5	7.6	6.0
***Clostridium spiroforme* DSM 1552**	8.6	7.2	1.4	0.6	7.3	2.9	3.5	4.1	7.9	11.8	3.7	0.9	2.3	3.3	2.6	11.5	0.5	4.2	7.7	8.2

COG categories

E, amino acid transport and metabolism
G, carbohydrate transport and metabolism
D, cell cycle control
N, cell motility
M, cell wall, cell membrane, and cell envelope biogenesis
H, coenzyme transport and metabolism
V, defense mechanisms
C, energy production and conservation
S, function unknown
R, general function prediction only

P, inorganic ion transport and metabolism
U, intracellular traffic, secretion and vesicular transport
I, lipid transport and metabolism
F, nucleotide transport and metabolism
O, post translational modification, protein turn over and chaperones
L, replication, recombination, and repair
Q, secondary metabolite synthesis, transport, and catabolism
T, signal transduction mechanisms
K, transcription
J, translation, ribosomal structure, and biogenesis
