# Supplementary material for: The Purine-Utilizing Bacterium Clostridium acidurici 9a: A Genome-Guided Metabolic Reconsideration
Source: PLoS One. 2012 Dec 11;7(12):e51662. doi: 10.1371/journal.pone.0051662 (PMC3519856; doi:10.1371/journal.pone.0051662)
Supplement: Table S3 — Genes encoding antibiotic resistance proteins of C. acidurici 9a. (PDF) [file pone.0051662.s006.pdf]

Table S3. Genes encoding antibiotic resistance proteins of *C. acidurici* 9a.

	Locus Tag	Annotation
**Vancomycin**	Curi_c05040	Putative vancomycin response regulator
	Curi_c05050	Vancomycin B/G-type resistance protein (VanW)
	Curi_c05060	D-alanine-D-serine ligase (VanG)
	Curi_c05070	D-alanyl-D-alanine carboxypeptidase (VanXY)
	Curi_c05080	Serine-type alanine/serine racemase (VanT)
	Curi_c20140	Vancomycin response regulator (VanR)
	Curi_c20130	Vancomycin sensor histidine kinase (VanS)
	Curi_c20120	D-alanyl-D-alanine carboxypeptidase (VanY)
	Curi_c20090	Putative D-alanyl-D-alanine carboxypeptidase
	Curi_c20080	VanZ family protein
**Ampicillin**	Curi_c00470	Penicillinase repressor (BlaL1)
	Curi_c00480	Beta-lactamase regulatory protein (BlaR1)
	Curi_c26710	Putative metallo-beta-lactamase
	Curi_c26740	Putative penicillinase repressor
	Curi_c26750	Putative beta-lactamase regulatory protein
	Curi_c27160	Penicillinase repressor (BlaL2)
	Curi_c27170	Beta-lactamase regulatory protein (BlaL2)
	Curi_c27730	Putative metallo-beta-lactamase
**Bacitracin**	Curi_c18920	Bacitracin resistance protein (BacA)
	Curi_c27290	Bacitracin ABC transporter permease protein (BcrA)
	Curi_c27280	Bacitracin ABC transporter ATP-binding protein (BcrB)
**Chloramphenicol/Thiamphenicol**	Curi_c07100	Chloramphenicol acetyltransferase (Cat1)
	Curi_c07640	Chloramphenicol acetyltransferase (Cat2)
	Curi_c15350	Chloramphenicol acetyltransferase (Cat39
**Erythromycin/Clarithromycin**	Curi_c02730	Putative macrolide ABC transporter ATP-binding protein
	Curi_c02740	Putative macrolide ABC transporter permease protein
	Curi_c00630	Putative macrolide exporter ATP-binding/permease protein
	Curi_c03050	Outer membrane efflux protein
**Acriflavine**	Curi_c03060	RND family efflux transporter, MFP subunit
	Curi_c03070	Acriflavine resistance protein
	Curi_c03080	TetR family response regulator
	Curi_c29460	Acriflavine resistance protein
	Curi_c29470	RND family efflux transporter, MFP subunit
	Curi_c29480	TetR family response regulator
	Curi_c06580	BC amino acid permease/azaleucin resistance protein (AzlC1)
**4-azaleucine**	Curi_c25360	BC amino acid permease/azaleucin resistance protein (AzlC2)
	Curi_c25250	BC amino acid transport protein/azaleucin resistance protein (AzlD)
