# Supplementary material for: The Purine-Utilizing Bacterium Clostridium acidurici 9a: A Genome-Guided Metabolic Reconsideration
Source: PLoS One. 2012 Dec 11;7(12):e51662. doi: 10.1371/journal.pone.0051662 (PMC3519856; doi:10.1371/journal.pone.0051662)
Supplement: Table S4 — Antibiogram of C. acidurici 9a. (PDF) [file pone.0051662.s007.pdf]

Table S4. Antibiogram of *C. acidurici* 9a.

Antibiotic	Concentration (µg/ml)	[1]Maximum OD_{600nm} (Ø)	Standard deviation
Vancomycin	5	0.44	0.02
	10	0.3	0.02
	20	0.09	0.009
	40	0.09	0.008
Ampicillin	20	0.3	0.02
	50	0.28	0.02
	100	0.19	0.01
	150	0.07	0.008
Bacitracin	10	0.69	0.02
	30	0.71	0.03
	50	0.69	0.01
	100	0.72	0.01
Chloramphenicol	5	0.54	0.03
	10	0.35	0.01
	30	0.23	0.02
	50	0.22	0.009
Thiamphenicol	2	0.51	0.02
	5	0.45	0.01
	15	0.33	0.02
	30	0.22	0.03
Erythromycin	5	0.36	0.009
	10	0.24	0.02
	50	0.1	0.01
	100	0.1	0.01
Clarithromycin	2	0.49	0.01
	5	0.41	0.02
	10	0.36	0.01
	20	0.2	0.02
Acriflavine	50	0.63	0.01
	150	0.62	0.02
	250	0.64	0.01
	350	0.61	0.02
4-azaleucine	30	0.63	0.02
	50	0.62	0.04
	100	0.63	0.01
	200	0.61	0.03
Kanamycin	20	0.09	0.009
	50	0.08	0.009
	100	0.08	0.02
	150	0.07	0.01
Gentamicin	10	0.07	0.01
	20	0.08	0.01
	50	0.08	0.008
	100	0.09	0.01
Tetracyclin	5	0.1	0.009
	10	0.08	0.009
	20	0.09	0.01
	30	0.09	0.01
Without antibiotic	-	0.7	0.03

[1]Tests were carried out in triplicate and the average OD_{600nm} was calculated
