# Supplementary material for: The Purine-Utilizing Bacterium Clostridium acidurici 9a: A Genome-Guided Metabolic Reconsideration
Source: PLoS One. 2012 Dec 11;7(12):e51662. doi: 10.1371/journal.pone.0051662 (PMC3519856; doi:10.1371/journal.pone.0051662)
Supplement: Table S5 — Genes encoding metal resistance proteins of C. acidurici 9a. (PDF) [file pone.0051662.s008.pdf]

Table S5. Genes encoding metal resistance proteins of *C. acidurici* 9a.

Accession No.	Annotation	Ions
Curi_c01370	Copper-translocating P-type ATPase (CopA)	Cu^{+}/Ag^{+}
Curi_c01380	Copper chaperone (CopZ)	Cu^{+}/Ag^{+}
Curi_c01390	Copper-sensing transcriptional regulator (CsoR)	Cu^{+}/Ag^{+}
Curi_c01850	Chromate transport protein (ChrA1)	Cr^{6+}
Curi_c01860	Chromate transport protein (ChrA2)	Cr^{6+}
Curi_c04760	Chromate transport protein (ChrA3)	Cr^{6+}
Curi_c06400	Cadmium-, zinc-, and cobalt-transporting P-type ATPase (CadA)	Zn^{2+}/Cd^{2+}
Curi_c26810	Cadmium, zinc, and cobalt/H^{+}-K^{+} antiporter (CzcD)	Zn^{2+}/Cd^{2+}
Curi_c11220	Cation-transporting P-type ATPase	Zn^{2+}/?
Curi_c15240	Putative aluminium resistance protein	Al^{3+}
